# Molecular Pathways Involved in the Anti-Cancer Activity of Flavonols: A Focus on Myricetin and Kaempferol

**DOI:** 10.3390/ijms23084411

**Published:** 2022-04-16

**Authors:** Maria Rosa Felice, Alessandro Maugeri, Giovambattista De Sarro, Michele Navarra, Davide Barreca

**Affiliations:** 1 Department of Chemical, Biological, Pharmaceutical and Environmental Sciences, University of Messina, 98166 Messina, Italy; mrfelice@unime.it (M.R.F.); amaugeri@unime.it (A.M.); davide.barreca@unime.it (D.B.); 2 Department of Health Sciences, University “Magna Græcia” of Catanzaro, 88100 Catanzaro, Italy; desarro@unicz.it

**Keywords:** cancer, flavonols, myricetin, kaempferol, in vitro, in vivo, polyphenols

## Abstract

Natural compounds have always represented valuable allies in the battle against several illnesses, particularly cancer. In this field, flavonoids are known to modulate a wide panel of mechanisms involved in tumorigenesis, thus rendering them worthy candidates for both cancer prevention and treatment. In particular, it was reported that flavonoids regulate apoptosis, as well as hamper migration and proliferation, crucial events for the progression of cancer. In this review, we collect recent evidence concerning the anti-cancer properties of the flavonols myricetin and kaempferol, discussing their mechanisms of action to give a thorough overview of their noteworthy capabilities, which are comparable to those of their most famous analogue, namely quercetin. On the whole, these flavonols possess great potential, and hence further study is highly advised to allow a proper definition of their pharmaco-toxicological profile and assess their potential use in protocols of chemoprevention and adjuvant therapies.

## 1. Introduction

Cancer is the second leading cause of death worldwide. In 2020, 19.3 million new cases of cancer were recorded, along with 10 million deaths caused by this disease [1]. Irregular lifestyle habits characterized by scarce physical activity and unbalanced diets are acknowledged to be among the most significant risk factors for cancer [2]. More specifically, cancer originates from mutations in genes encoding for growth and transcription factors, protein kinases, apoptotic signaling proteins, or adhesion molecules. These initiated cells thereby acquire irreversible genetic alterations that are kept with each subsequent round of proliferation. This second stage of carcinogenesis, known as promotion, is a relatively long and reversible process, which comes from genetic and epigenetic modifications, causing selective clonal expansion. During promotion, cells develop the capacity to resist planned apoptosis and immune control, while maintaining angiogenetic capabilities. The majority of cancer cells remains elusive until the third stage of progression, when the tumor has already become malignant and ready to eventually spread to other parts of the body [3]. Therefore, cancer development arises from the progressive concatenation of several events. For this reason, agents aiming simultaneously at different targets represent an optimal strategy to counteract cancer.

In this field, compounds from natural origins have always represented valuable allies in the battle against several illnesses, especially cancer [4,5]. In particular, flavonoids are known for their undoubted beneficial properties; indeed, polyphenol-rich foods, consumed daily, can help the human organism to counteract an exaggerated inflammatory and oxidant status, as can be found in infections, auto-immune and neurodegenerative diseases, as well as in cancer [6,7,8,9,10,11,12]. Regarding the latter, flavonoids modulate a wide array of mechanisms involved in tumorigenesis [13,14,15,16]. Interestingly, it has been shown that flavonoids are able to hamper cancer development from both genetic causes [17], and also external causes (i.e., pollution, smoking or radiation) [18]. The main mechanism through which flavonoids act to achieve these effects is by scavenging reactive oxygen and nitrogen species, mainly via chelating metallic ions [19]. These species are also present during inflammation [20,21], and flavonoids are able to target intracellular factors such as nuclear factor kappa B (NF-κB), mitogen activated protein kinases (MAPKs), cyclooxygenase-2 (COX-2) and sirtuin 1 (SIRT1) [22,23,24,25]. The suppression of both chemokines and cytokines, via immune cell regulation, is another mechanism through which flavonoids act as anti-cancer agents, since these elements are involved in both cancer progression and spreading [26]. Flavonoids have been demonstrated to actively suppress cancer metastasis factors by modulating adhesion molecules including metalloproteinases and other epithelial-mesenchymal transition signals [27,28,29]. Angiogenesis is another important process in cancer progression and migration, as it is crucial for the effective sustenance of the tumor microenvironment. As a result, one of the routes followed by these compounds to combat cancer formation is to inhibit key factors in this process, such as vascular endothelial growth factor (VEGF) or epithelial growth factor receptor (EGFR) expression [30,31]. The escape from apoptosis represents one of the main characteristics acquired by cells becoming cancerous, and flavonoids are able to interfere with this process by inhibiting the activity of caspases and Bcl-2 family members [32,33,34]. Activation of beclin-1 and microtubule-associated proteins 1A/1B light chain 3B (LC3), markers of the early and late phases of autophagosome formation, respectively, can help flavonoids repair defective autophagy in tumor cells [35]. Furthermore, altered cell cycle progression is another important factor in tumor growth, and flavonoids have been extensively studied in this area, with their potential to affect the expression of numerous cyclin isoforms engaged in each phase of the cell cycle being discovered [36]. Among flavonoids, flavonols are, by far, the most abundant derivatives, and the ones showing the most interesting properties, such as the promotion of epigenetic changes and the Ubiquitin-proteasome pathway [37,38,39,40].

**Figure 1:**
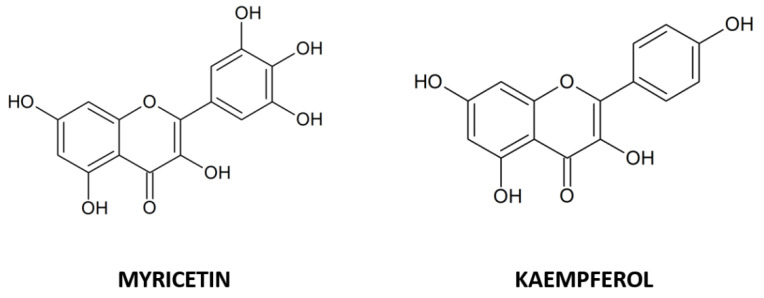
Chemical structures of the flavonols myricetin and kaempferol.

Currently, among the dietary flavonols, quercetin is in the spotlight for its undoubted anti-cancer properties. Nonetheless, other analogues have proved to possess outstanding capabilities. Therefore, in this review, we have focused our attention on myricetin and kaempferol (Figure 1), two of the most abundant dietary flavonols in natural matrices. They are characterized by specific chemical substituents present in the basic skeleton of flavonols and interact selectively with specific intercellular signalling pathways to induce different processes inside the cells, which have shown evidence of anti-cancer effects in different in vitro and in vivo models.

## 2. Myricetin

### 2.1. Myricetin and Hepatocarcinoma 

Among flavonols, the anti-tumoral effect of myricetin has been object of study for several years and its beneficial properties were immediately evident. First studies using the HepG2 cell line as a model, have highlighted that myricetin treatment induced apoptosis and blocked cells in the G2/M phase [41] at the concentration of 66 µM, and this effect was related to the decrease of cyclins A1 and B1, cyclin-dependent kinase 1 (CDK1) and CDK7, and to the increase of p21, p27 and p53 protein levels [42]. From these first promising results, several researchers have focused their attention on studying the effects of the molecule in different cell lines and in vivo, trying to define the mechanisms at the basis of these observations. A complex pathway, for example, was discovered using an animal model of hepatocellular carcinoma (HCC) and HepG2 cell line treated with myricetin 100 mg/Kg or 20 µM, respectively. It was demonstrated that it could reverse the increase of extracellular signal-regulated kinases (ERK1/2), protein kinase B (AKT), p21- activated kinase 1(PAK1) phosphorylation and the upregulation of different proteins, such as survivin, proliferating cell nuclear antigen (PCNA), Cyclin D1 and B-cell lymphoma 2 (Bcl-2), probably by a direct binding to PAK1, as highlighted by docking analysis, that alters the structural and functional properties of the kinase [43]. Using the same cell line, it was also demonstrated that myricetin (132 and 198 µM) treatment induced apoptosis by a mechanism in which the authors observed a decrease of phosphorylated AKT and p70S6K1 that led to an increase of the level of Bcl-2 associated agonist of cell death (BAD) protein [44]. Moreover, treatment of HepG2 and Huh-7 cell lines led to the discovery of the anti-proliferative capacities of myricetin (100 or 200 µM) that were linked to the effect on the Hippo pathway. In particular, myricetin enhanced the catalytic activity of large tumor suppressor kinase 1/2 (LATS1/2) that phosphorylates yes-associated protein 1 (YAP1) protein leading to its cytoplasmic sequestration and proteasomal degradation. Moreover, myricetin increased cisplatin anti-cancer activity both in vitro and in HCC xenograft mice, using the same molecular mechanism [45]. Beyond the anti-proliferative properties of myricetin, it can also negatively influence the epithelial mesenchymal transition (EMT) process, as demonstrated using the MHCC97H cell line; in fact, myricetin, at the dose of 100 µM, had a strong inhibitory capacity on migration and invasiveness, and this effect seems to be related to an increase of E-cadherin and a decrease of N-cadherin and vimentin levels, proteins that have different effects on cellular proliferation and cell-cell adhesion [46,47]. Iyer et al., (2015) demonstrated that myricetin inhibits PAK1 activity [43], a kinase that was found dysregulated or hyper-activated in HCC [48] and, for this reason, it could be considered the key protein target of myricetin. For example, PAK1 can phosphorylate Merlin (NF2) of the Hippo pathway, reducing its dual binding capacity with YAP1 and LATS1/2, preventing YAP phosphorylation and permitting its nuclear translocation [49]. Moreover, PAK1 is also implicated in AKT activation, facilitating its recruitment on plasma membranes by PDK1 (3-phosphoinositidedependent kinase-1), which is responsible of the first phosphorylation event necessary for AKT activation [50]. A possible involvement of PAK1 in EMT can be also be considered as it has been demonstrated that it can stimulate nuclear translocation of NF-κB, which is responsible for transcription of twist-related protein 1 (*TWIST1*), snail family transcriptional repressor 2 *(SNAI2*) and Smad interacting protein 1 (*SIP1*) genes, whose protein products, Twist1, Snail2 and Sip1, are a transcriptional repressor of E-cadherin and activator of N-cadherin [51,52,53,54,55,56].

### 2.2. Myricetin and Colorectal Cancer 

Among the different typologies of tumors, colorectal cancer (CRC), which is very aggressive and has a poor prognosis, represents the third most common type of tumor in the world for females, after breast and uterine cancers, and the second for males, after prostate cancer [57]. For this type of cancer, myricetin effects have been studied principally using cell lines and, at present, there are very few data from in vivo experiments. Moreover, the in vitro results were not able to delineate a common response to myricetin. First of all, there is a great discrepancy in the concentration used to inhibit growth, and it is not clear if myricetin is able to induce apoptosis. For example, in Caco-2 and HT-29 cell lines, the IC_50_ values for myricetin growth inhibition were 88.4 ± 3.4 and 47.6 ± 2.3 µM, respectively; the Caco-2 cells remained totally viable [58], whereas, in the HCT-15 cell line, there was 70% reduced viability and apoptosis observed at 100 µM myricetin concentration. In respect of the normal apoptotic proteins profile, the authors did not observe an increase of caspase-3, -9 or mitochondrial cytochrome C release, but only an increase of apoptosis inducing factor (AIF) release that is responsible for a caspase-independent apoptosis pathway [59]. Using the same cell line, in the same experimental conditions, an increase of cleaved caspase-3, -9 and poly (ADP-ribose) polymerase 1 (PARP1) was observed and the effect was linked to an increase of nucleoside diphosphate kinase (NDPK) expression, as highlighted by proteomic analysis conducted by 2D electrophoresis followed by MALDI-TOF. The involvement of NDPK in inhibition of metastasis and induction of apoptosis was confirmed by cell knockdown, in which the apoptosis rate was reduced [60]. Differently, in the COLO 205 cell line, 200 µM myricetin had only a slight cytotoxic effect and no DNA fragmentation was observed [61], whereas the HCT116 cell line used in another study was irresponsive to myricetin, even when cells were exposed to a concentration > 500 µM [62]. HCT116 and other cell lines (HT-29, SW480, and SW620) that were not responsive to myricetin in the same manner have highlighted that the treatment with flavonol (range 0–400 µM) induced autophagy and apoptosis by inhibiting the phosphatidylinositol 3-kinase (PI3K)/AKT/ mammalian target of rapamycin (mTOR) pathway; furthermore, the level of apoptosis increased when autophagy was inhibited, indicating that co-administration of autophagy inhibitor drugs with myricetin can enhance its cytotoxic effects [63]. In this last study and in the above mentioned, however, it is evident that the cell lines used do not respond in the same manner to myricetin, and this different behavior must be ascribed to the different mutations in essential genes [64], suggesting that the genotype and, eventually, epigenotype of cancer cells, even if representative of the same cancer model, can be a variable to consider more deeply. Another problem in the use of myricetin in colorectal cancer is to define the protein/pathway targets. In the COLO 205, COLO 320HSR, COLO 320DM, HT-29, and COLO 205-X cell lines, myricetin (200 µM) had a strong inhibitory effect on secreted metalloproteinase-2 (MMP-2) protein activity (90% inhibition) and its expression was also negatively regulated by a decrease of ERK1/2 and c-Jun phosphorylation, and protein kinase C alpha (PKCα) membrane translocation, indicating that myricetin, in addition to direct inhibition of MMP-2 activity, probably regulates its expression acting on the PKCα pathway [61]. In this study, PKCα is considered an oncogene, but recent research has highlighted that PKCα overexpression leads to an enhanced rate of cancer cell death, probably by a mechanism in which PKCα inhibits β-catenin function, suggesting a tumor suppressor function for this kinase in CRC, where its expression is very low compared to normal tissue [65]. 

Myricetin, with an IC_50_ of 690 nM, is also able to inhibit in vitro the activity of human flap endonuclease 1 (hFEN1) protein that is highly expressed in colon cancer cells and is associated with poor prognosis. Even if myricetin is not able alone to induce cell death at the relatively low concentrations tested (0–64 µM), the inhibition of hFEN1 could enhance the cytotoxicity of paclitaxel, a chemotherapeutic molecule that causes DNA double strand breaks [66]. Another important protein whose activity can be negatively regulated by myricetin is multidrug resistance-associated protein 2 (MRP2), which is highly expressed in CRC. The administration of myricetin at a concentration of 60 µM to the Caco-2 cell line, was able to decrease the oxaliplatin drug efflux by MRP2 and thereby promote the drug accumulation and apoptosis rate [67] probably by direct binding of the flavonol to the transporter, competing for its natural substrate and inhibiting its ATPase activity [68,69]. Myricetin was also tested as a chemopreventive molecule in APC^Min/+^ mice, a model of familial adenomatous polyposis [70]. The oral administration of myricetin (100 mg/kg) was able to decrease the size and number of polyps by cellular growth inhibition and apoptosis induction. A deeper analysis has revealed that the treated mice presented an increase of active non-phosphorylated glycogen synthase kinase 3 beta (GSK-3β) and an increase of the destabilized form of phosphorylated β-catenin (Ser37) levels, indicating that myricetin, through GSK-3β, inhibited β-catenin activation, probably inhibited both expression and phosphorylation of c-Jun N-terminal kinase (JNK), p38/MAPK, ERK1, as well as AKT and mTOR phosphorylation. Moreover, a decrease of inflammation was also observed [71], and the same result was obtained by another study conducted using a more severe mouse model of colonic colitis. It was found that myricetin (100 mg/kg) administration decreased cytokines production, and expression of NF-κB and COX-2. This effect was probably linked to the reduction of tumor necrosis factor alpha (TNF-α) expression, derived from β-catenin degradation [72].

### 2.3. Myricetin and Breast Cancer

Myricetin was also tested as chemotherapeutic molecule in different breast cancer cell lines. Triple-negative breast cancer (TNBC) represents 15% of total breast cancer cases. In human TNBC cell line, MDA-MB-231, myricetin reduced cell growth (IC_50_ = 114.75 µM after 72 h) by induction of apoptosis [62]. These results were partly confirmed for the same cell line and extended to MDA-MB-468, as well as ER^+^ MCF-7 and human epithelial growth factor receptor 2 (HER2)-overexpressing SK-BR-3 that showed growth inhibition at 50 µM myricetin concentration (80%) with a very similar efficacy to doxorubicin. The effect was linked to increased reactive oxygen species (ROS) production as a consequence of the formation in the growth medium of H_2_O_2_, which enters inside the cells where it reacts with iron, via the Fenton reaction. ROS production led to mitochondrial membrane depolarization, release of cytochrome C and an increase of gamma H2A histone family member X (γH2AX) phosphorylation, effects that were reverted by N-acetyl-cysteine treatment but not by caspase or necroptosis inhibitors, indicating the involvement of a caspase-independent mechanism, for example by AIF mitochondrial release. Moreover, the researchers also observed an increase of ERK1/2 and p38/MAPK phosphorylation, an effect linked to stress conditions rather than to involvement in induction of apoptosis, as demonstrated by the use of kinase inhibitors [73].

In MCF-7, it was observed that telomerase is overexpressed and treatment with 50 µM myricetin repressed human telomerase reverse transcriptase (*TERT*) gene expression of about 90%. This effect could be linked to the inhibitory activity of myricetin on different pathways that regulate c-Myc activity, a transcription factor that directly controls human *TERT* gene expression [74]. It is noteworthy that myricetin in vitro is also able to bind to a G-quadruplex telomeric structure and stabilize it, thereby preventing telomerase binding and elongation of telomeres [75]. In the same cell line, 54 µM of myricetin was able to reduce cell viability by induction of extrinsic and intrinsic apoptosis pathways, and the anti-proliferative effect was also demonstrated by the increase of *TP53*, breast cancer type 1 susceptibility protein (*BRCA1*) and growth arrest and DNA damage-inducible protein 45 (*GADD45*) gene expression [76]. Very similar results were also obtained by the use of a T47D cell line in which myricetin treatment at 46 µM concentration increased the apoptosis rate and reduced cell viability by induction of the same genes except for *TP53*, which is mutated and not functional in this cell line, indicating that activation of programmed cell death by myricetin can be independent of p53 functionality and cellular genotype [77]. Induction of apoptosis was also demonstrated in MCF-7 by 80 µM myricetin treatment that was able to inhibit cell growth significantly by inducing caspase-mediated apoptosis. This effect was dependent on a decrease of ERK1/2 phosphorylation and of PAK1, MEK1 and β-catenin protein level, whereas an increase of GSK-3β expression was observed [78]. The observed reduction of the master regulator PAK1 level could be linked to induction by myricetin of its degradation by autophosphorylation, which is stimulated by interaction with the Rho-family GTPase proteins, calcineurin B homologous protein 1 (Chp) and cell division control protein 42 homolog (Cdc42) [79]. This could explain all the protein profile variation observed except for the MEK1/2 decreased protein level that could be linked to direct cleavage by activated caspase-3 [80].

**Figure 2:**
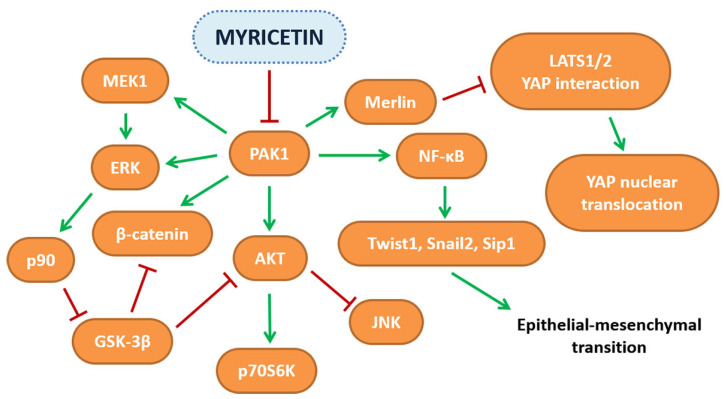
Schematic representation of the pathways affected by myricetin in several in vitro and in vivo cancer models.

One consequence of breast cancer is that it can lead to metastasis in the lungs and brain. To investigate if myricetin can exert beneficial effect also in this process, breast cancer brain metastasis (BCBM) MDA-Mb-231Br and mouse breast cancer 4T1 were used. Myricetin was able to inhibit growth at 40 µM concentration, and to reduce, at lower concentration, cell migration, invasiveness and adhesion. The authors of this study explained the results were associated with a reduction of the cellular hallmarks linked to metastasis formation including a decreased mRNA, protein level and activity of MMP-2 and -9. Moreover, *ST6GALNAC5*, a brain-specific gene that promotes brain metastasis formation by facilitating crossing of the blood brain barrier (BBB), was also reduced. The results obtained in vitro were partly demonstrated in vivo by using a 4T1 lung metastasis animal model in which the administration of 25 mg/Kg of myricetin led to the reduction of the number of tumor nodules [81]. Another potential target of myricetin is ornithine decarboxylase (ODC), a rate-limiting enzyme in polyamine synthesis and a molecule that is positively involved in tumorigenesis at different levels [82,83]. It was demonstrated that in breast cancer, inhibition of cellular polyamine synthesis and uptake has antiproliferative effects [84,85], and this discovery has led to the research of new molecules with inhibitory ability towards ODC. Even though myricetin was not tested directly on breast cancer cells, it was demonstrated that the flavonol can inhibit ODC activity with an IC_50_ of 7.3 µM, a dose 10-fold less concentrated in respect to α-DL-difluoromethylornithine (DFMO), an FDA-approved anti-cancer drug based on ODC inhibition. Moreover, molecular docking studies have revealed that myricetin binds near the active site at the dimerization interface and can induce apoptosis by acting directly on ODC [86]. Even if the in vitro results have highlighted the potential of myricetin, its use in vivo is limited by its low water solubility [87] and instability. One strategy that has been used to overcome these limitations is the encapsulation of myricetin in nanoparticles formed of BSA conjugated with folic acid. This system has the scope to target cancer cells that normally express a higher quantity of folate receptors on their membrane compared with normal cells. Using the MCF-7 cell line, a higher ROS production and apoptosis rate was observed when encapsulated myricetin was used compared with the free form, indicating the great importance of developing more efficient drug delivery systems [88]. An increase in cellular ROS production was also observed in the same cell line when gold nanoparticles with myricetin (Myr-AuNPs) were used, showing an IC_50_ of 13 µg/mL. ROS overproduction, an increase in chromosome DNA condensation, apoptotic bodies and mitochondrial membrane depolarization were also reported, indicating that gold nanoparticles represent a more efficient delivery system, both in terms of the myricetin concentration needed to inhibit cellular growth and in the increase of flavonol stability in the different physiological conditions [89]. Moreover, compared with free-form myricetin, myricetin encapsulated in nanostructured lipid carriers (NLC) and tested alone or in combination with the chemotherapeutic molecule, docetaxel, on MDA-MB-231 cells was demonstrated to be more effective in inducing apoptosis and arresting cell cycles in the sub-G1 phase and, moreover, enhanced the anti-cancer activity of docetaxel [90]. The pathways modulated by myricetin are depicted in Figure 2, while the scientific evidence on the anti-cancer activities of myricetin described in this review is reported in Table 1.

## 3. Kaempferol

### 3.1. Kaempferol and Hepatocarcinoma 

HCC is characterized by alteration of different pathways that lead to dysregulation of normal cellular physiology. Among the altered factors in HCC, hypoxia-inducible factor 1-alpha (HIF-1α) is overexpressed in this type of tumor and is related to its severity. Using the Huh7 cell line, grown in hypoxic conditions, it was demonstrated that kaempferol (IC_50_ = 4,75 µM) was able to inhibit HIF-1α ERK1/ERK2-dependent nuclear translocation [91]. In addition to HIF-1α, 40 µM kaempferol cell treatment led to upregulation of p53-inducible gene 3 (PIG3) expression, which is involved in apoptosis triggered by increased ROS production, resulting, according to the authors, from kaempferol autoxidation enhancement [92]. In support of kaempferol mediated ROS generation, Seydi et al., using hepatocytes isolated from an HCC rat model, demonstrated that kaempferol decreased cell viability (IC_50_ = 30 µM) by apoptosis, which was triggered by an increase of intracellular H_2_O_2_ [93]. The SK-HEP-1 cell line was also sensitive to kaempferol-mediated growth inhibition (IC_50_ = 100 µM), and the decrease observed was linked essentially to two different phenomena: blocking of cell cycle progression in G2/M and induction of autophagic cell death. This response could be linked to increased adenosine monophosphate-activated protein kinase (AMPK) activity that inactivates the mTOR pathway, a negative regulator of autophagy [94]. A relation between kaempferol and AMPK activity was also demonstrated using other HCC cell lines, such as HepG2, Huh7, BEL7402, SMMC and primary human HCC cells. Fifty µM of flavonol treatment decreased cell viability by inducing autophagic cell death. This effect was probably linked to increased AMPK phosphorylation and protein level, most likely dependent on downregulation of melanoma-associated antigen A6 (*MAGEA6*) expression, a specific AMPKα1 ubiquitin ligase [95]. In contrast with the above study, treatment of HepG2 cell line with 100 µM kaempferol decreased cell viability by triggering apoptosis after endoplasmic reticulum (ER) stress response, an effect that was reversed by its inhibition or DNA damage-inducible transcript 3 (*DDTI3*), encoding for C/EBP homologous protein (CHOP), silencing [96]. These results seem to be contradictory, but the study of Guo et al., using HepG2 and Huh7 cell lines, has demonstrated that both autophagic and apoptotic death are responsible for the minor cells viability after treatment with 100 µM kaempferol since inhibition of autophagy, triggered by endoplasmic reticulum (ER) stress, increases cell viability and decreases apoptosis rate, demonstrating that autophagy precedes apoptosis and highlighting that both mechanisms are involved in the beneficial effects exerted by kaempferol [97]. 

Apart from the effect exerted on cell viability, it was also demonstrated that the flavonol inhibits cellular migration and invasion when Huh-7 and SK-Hep-1 cell lines were used as a model, even if no evident cytotoxic effect was observed at the concentration tested (100 µM). The researchers observed a reduction of cathepsins and MMP-9 protein levels which was probably linked to the kaempferol-mediated decrease of AKT phosphorylation, suggesting that this kinase can be involved in the metastasis process [98]. Given the positive effect of kaempferol on cellular responses, it was decided to use it in combination with chemotherapeutic molecules, such as sorafenib, against which HCC is highly resistant. HepG2 and Hep3B cell lines pre-treated with kaempferol or treated with a combination of kaempferol and sorafenib, exhibited lower IC_50_ values relative to growth inhibition in respect to sorafenib used singularly, an effect related to increased apoptosis rate [99]. In silico docking studies have revealed a high score of interaction for kaempferol with multidrug resistance protein-1 (MDR-1), and this interaction was probably the basis of the major cytotoxic effect observed in N1S1 and HepG2 cell lines when a combination of oxaliplatin, at sub-lethal concentration (2.5 µM), and kaempferol (2.5 µM), were used, highlighting an important field of application against cancer chemoresistance and dose toxicity [100]. The combination of kaempferol and doxorubicin was also considered, and when tested on different liver cancer cell lines, an additive effect of the two molecules was demonstrated in respect of viability, growth inhibition, apoptosis rate, colony formation, migration and invasiveness processes. This effect could be mediated by decrease of AKT, PI3K, mTOR, and ribosomal protein S6 kinase (S6K) protein levels that were found both in treatment with kaempferol and doxorubicin alone, and more markedly, when the two substrates were used in combination [101]. 

### 3.2. Kaempferol and Colorectal Cancer 

The first studies focusing on the beneficial effects of kaempferol against colorectal cancer have produced contrasting results. For example, the SW480 cell line was sensitive to flavonol-mediated growth inhibition (IC_50_ = 100 µM), even if low rate of apoptosis was evident [102], whereas in HT29, COLO205, COLO320-HSR, and COLO205-X, the use of 200 µM of kaempferol was ineffective on cell viability [103]. Over the years, more accurate methodologies were used and the multiple beneficial effects of kaempferol against this kind of cancer were in part defined, and they involve target proteins as well as signaling pathways, although the majority of the research has been focused on *in vitro* studies. In the SW480 cell line, a 50 µM kaempferol treatment that led only to slight apoptosis, was able to inhibit the activity of the pro-inflammatory and tumor promoting 12-(S)-lipoxygenase when it was overexpressed under the control of a cytomegalovirus promoter, indicating a direct effect of the flavonol on enzyme activity [104]. At a concentration determined as non-cytotoxic for cells, 40 µM kaempferol could reduce stimulated TNF-α COX-2 expression, a protein related to carcinogenesis, in a DDL-1 cell line and the reduction of its basal expression level was evident also in absence of the cytokine [105]. Another protein, whose expression was upregulated by 40 µM kaempferol in SW480 cell line, is organic anion/cation transporter 2 (OCTN2), a protein involved in the uptake of different molecules such as oxaliplatin that is transcribed by the heterodimer peroxisome proliferator-activated receptor/retinoid X receptor factor (PPARγ/RXR). The mechanism proposed was a direct interaction of kaempferol with PPARγ, as predicted by molecular docking analysis, that by increasing OCTN2 expression, augmented the uptake of oxaliplatin and its cytotoxicity, thereby ameliorating the efficacy of the chemotherapeutic molecule [106]. Apart from OCTN2, kaempferol can regulate expression of DR5 (Death Receptor 5), which is involved in extrinsic apoptosis, as demonstrated using SW480 and DLD-1 cell lines. 40 µM kaempferol, a non-cytotoxic dose, in combination with TNF-related apoptosis-inducing ligand (TRAIL) ligand, enhanced apoptosis by upregulating death receptor (DR) 4 and DR5 expression, suggesting the role of kaempferol in this process [107]. In contrast to the above reported results, in a HCT116 cell line that possesses the *TP53* wild-type gene [108], kaempferol treatment led to a decrease in cell viability (IC_50_ = 53.6 µM) by induction of intrinsic apoptosis. This was triggered by ataxia-telangiectasia mutated (ATM) that is activated after DNA damage, and positively regulates p53 activity [109]. Moreover, in some colorectal cancer tissues, as in the LoVo cell line, studies have reported an inactivating frameshift mutation in the *BAX* gene that make cells relatively resistant to apoptosis and can be at the base of chemoresistance in familial colorectal cancers. Using a Bcl-2-associated X protein (*BAX*) knock-out HCT116 cell line, but confirmed also in LoVo cells, it was demonstrated that 100 µM kaempferol induced apoptosis, probably by activation of the Bcl-2 homologous antagonist killer (Bak) pro-apoptotic protein as consequence of an ER stress response, indicating a general efficacy of kaempferol even when cells present different genotypes [110]. In the landscape of the genetic heterogeneity of colon cancer cell lines, in HT-29 and SW480 that have the *TP53* mutant gene [108], kaempferol induced apoptosis in a dose-dependent manner (0–60 µM) by decreasing phosphorylated AKT levels that corresponded to the activation of different proteins involved in intrinsic apoptosis. Moreover, AKT can indirectly regulate transcription of Fas ligand indicating that kaempferol can also stimulate an extrinsic apoptosis pathway [111]. Apart the genetic variability, another very important factor to consider is the epigenetic aspect in that 15–20% of CRC can present CpG island methylated phenotypes, and alterations were discovered also in the pathway related to WNT/β-catenin. In particular, dishevelled binding antagonist of beta catenin 2 (DACT2) that binds to β-catenin preventing its transactivator activity presents, in many CRC, a hypermethylated promoter that correlates with lower expression in tumor tissue compared with normal tissue. Using HCT116 and HT-29 cell lines, it was highlighted that kaempferol (5 µM) increased apoptosis and necrosis, and reduced cell migration capacity by upregulating DACT2 expression, a phenomenon that correlated with a hypomethylation state of its promoter and was probably linked to decreased DNA methyl transferase (DNMT) 1, 3a and 3b protein levels. Concerning the influence on DNMTs expression, in silico docking studies have revealed that kaempferol can bind to the catalytic site of DNMT1, probably interfering also with its activity. The in vitro results were partially confirmed in vivo using an animal model of induced CRC; in fact, oral administration of 150 mg/kg kaempferol led to an increase of DACT2 expression with concomitant downregulation of β-catenin transcribed genes, and this was reflected in a colon normal load and a decrease of nodules [112]. Using SW480, HCT116, and HCT-15 cell lines, it was demonstrated that kaempferol (IC_50_ = 50 µM) induced apoptosis and this effect seemed to be related, mostly for HCT116 and HCT-15 cell lines, to increased ROS production that led to increased levels of p53 and phosphorylated p38/MAPK, whereas a decrease in phosphorylated ERK1/ERK2 and JNK was observed. This indicates a cross-talk between p53 and p38/MAPK activity, probably activated by increased kaempferol-mediated ROS production [113]. An alarming complication is that CRC often acquires resistance toward chemotherapeutic molecules such as 5-fluorouracil (5-FU), a molecule that targets thymidylate synthase enzyme and is still extensively used today. In the study of Riahi-Chebbi et al., the authors developed a colon cancer cell line resistant to 5-fluorouracil, LS174-R characterized by increased expression of ATP-binding cassette (ABC) transporters, an EMT phenotype and high levels of phosphorylated ERK1/ERK2, p38 and AKT. Using a non-cytotoxic concentration of 5-FU, the addition of 75 µM kaempferol was able to induce apoptosis and to block cells in S phase, demonstrating a synergistic effect between the two molecules. Differently to other studies, they found a reduction of ROS production in the same experimental conditions. At a molecular level, the combined treatment reduced the level of p38α and AKT phosphorylation, and enhanced ERK1/ERK2 phosphorylation, demonstrating a correlation between these pathways. In support of the role of p38 in the cellular response is the probable inhibition of p38α activity by kaempferol interaction, as was suggested by molecular docking analysis. Moreover, in the same experimental conditions, the researchers discovered increased p53 phosphorylation, a reduction of STAT3, NF-κB, VEGF and interleukin-8 protein levels, the last two implicated in angiogenesis, and thymidylate synthase, that is normally overexpressed in LS174-R, also resulted in downregulation in combined treatment [114]. Similar results were obtained using HCT116 and HCT-8 cell lines, which show different IC_50_ in respect to kaempferol and 5-FU. HCT-8, which has higher IC_50_ values (kaempferol IC_50_ = 177.78 µM and 5-FU IC_50_ = 350 µM), was used at a lower concentration in respect to IC_50_ (50 µM 5-FU and 100 µM kaempferol), and the two molecules showed a synergistic effect in inducing apoptosis. In this case it is also noteworthy that thymidylate synthase protein level was reduced when the two molecules were used in combination. This occurred by activity reduction of the PI3K/AKT pathway that is involved in resistance towards 5-FU and in regulation of thymidylate synthase expression [115]. Recently, it was demonstrated that kaempferol exerts its beneficial effect by repression of aerobic glycolysis, the preferred form of energy production in cancer cells. Studies at the molecular level, using HCT116 and DLD-1 cell lines, have highlighted the involvement of miR-339-5p, upregulated by kaempferol, that target two different mRNAs, heterogeneous nuclear ribonucleoprotein A1 (hnRNPA1) and polypyrimidine tract-binding protein 1 (PTBP1) that, by alternative splicing of *PKM* mRNA, lead to M2 type-pyruvate kinase (PKM2) production, overexpressed in cancer cells compared with PKM1 expressed in normal cells. Using different experimental strategies, the researchers demonstrated that the downregulation of PKM2 led to a decrease of glycolysis that led directly to induction of apoptosis, growth inhibition, and colony formation [116]. In the literature analyzed in this review, only very few articles carried out experiments based on omics sciences, at least for the two flavonols examined in this context. Moreover, different genes differently expressed are reported, and the experimental approaches are very different. These are related to beneficial effects of kaempferol in CRC. For example, a microarray dataset of 17 human colorectal cancer tissues and 17 matched normal tissues were examined for differential gene expression, and among these, four potential gene derived proteins (prostaglandin-endoperoxide synthase 2 -*PTGS2*, nuclear receptor subfamily 3 -*NR3- C2* and *CA2*, as well as *MMP-1*) showing a high score of interaction with kaempferol were identified by molecular docking, but the authors did not validate the results by in vitro or in vivo studies [117].

The approach of Zhou et al. was different. These authors analyzed three distinct public human transcriptomic datasets from colorectal cancer and normal tissues and identified a panel of genes differently expressed. The corresponding 3D protein structures were screened for interaction with different molecules, as with Kaempferol, by molecular docking. Among these, a high score was registered between kaempferol and cyclin D1 (*CCND1*) and p65 (*RELA*), NF-κB subunit. In the attempt to confirm the prediction data, HCT116 and LoVo cell lines were treated with flavonol at concentrations between 0 and 120 µM. The authors reported Bcl-2, *RELA* decreased and Bax increased expression that was reflected in an increased apoptosis rate, indicating mainly kaempferol-mediated regulation rather than binding inhibition, as confirmed by overexpression of *RELA* that reverts the expression gene profile observed in vitro [118].

Another study was based on HCT116 and RKO cell lines treated with low concentrations of kaempferol (9.427 and 17.42 µM, respectively) that induced cell cycle arrest and apoptosis. Transcriptomic analysis revealed a pattern of at least 50 genes, including coding and noncoding RNA, up and down regulated, respectively, involved in different cellular pathways. Moreover, genomic analysis revealed that kaempferol induces point mutations in colony stimulating factor 1 receptor (*CSFR1*; in untranslated region). One in *TP53* is not malignant and one in phosphatidylinositol-4,5-bisphosphate 3-kinase (*PIK3CA*) reverts the malignant mutation in the RKO cell line, whereas in HCT116, only one nucleotide variation that abolishes a pathogenic mutation was reported in the c-*KIT* gene. Although these results are very interesting and open new research perspectives based on differential gene expression, the authors did not investigate their biological significance in more detail. Moreover, the point mutations introduced in the genome after kaempferol cell treatment that are different in the different cell lines further highlight how much is still unknown about the mechanism of action of these molecules and their impact on cell metabolism [119].

### 3.3. Kaempferol and Breast Cancer

In breast cancer, the inhibitory response to kaempferol is dependent on the typology of the cancer cells examined because the presence or absence of the characteristic receptors that are used for their classification (estrogen receptor, progesterone receptor, human EGFR2) can influence not only the concentration but also the cell mechanism used to respond to the stimulus, and moreover, breast cancer cells have other mutations in essential genes that can alter the response, justifying the different results reported in literature. For these reasons, the molecular characteristics of cell lines must be considered in more detail. In particular, the study of Hung demonstrated that MCF-7, T47D, and ZR-75 (ER^+^), MDA231 (ER^−^) were differently sensitive to kaempferol-mediated growth inhibition (IC_50_ values 35 µM and 70 µM, respectively), and this observed effect was linked, in ER^+^ cells, to ERα reduced protein level caused by induction of its proteasomal degradation and abolishment of the β-17-estradiol (E2)-mediated proliferation [120]. This was in turn due to the antagonizing nature of kaempferol in respect to E2 in binding to ERα, as predicted by molecular docking [121] and confirmed in VM7Luc4E2, an engineered breast cancer cell line derived from MCF-7, in which treatment with low concentrations of kaempferol (30–40 µM) induced apoptosis, antagonizing the proliferative effect not only of E2, but also of Triclosan (TCS) and bisphenol A. The mechanism of this process was kaempferol-mediated ROS production that led to an ER stress response, as demonstrated by increased CHOP and phosphorylated eukaryotic translation initiation factor 2 alfa (eIF2α) levels [122]. Instead, in SK-Br-3 breast cancer cells (ER^−^, PR^−^, HER2^+^) defective in Bak, treatment with 100 µM of kaempferol, that was demonstrated to causes ER stress, was ineffective in inducing apoptosis, and the cell survival was guaranteed by autophagy, indicating that apoptosis mediated by ER-stress, requires a functional Bak protein [110]. In any case, it is not to be ignored that the *TP53* gene in SK-Br-3 cells is partly functional and this can negatively influence the apoptosis process.

The anti-proliferative kaempferol-mediated effect in respect to triclosan and E2 was confirmed by other researchers using an MCF-7 and breast cancer xenograft mouse model. In the attempt to define the molecular mechanism, the authors considered the insulin-like growth factor 1 receptor (IGF-1R) pathway and discovered that E2 or TCS treatment led to increased insulin receptor substrate 1 (IRS1), AKT, MEK and ERK1/ERK2 phosphorylation, as well as increased expression of proteins involved in cell cycle progression, effects that were abolished by co-treatment with 50 µM kaempferol. This result suggests a connection between E2, ER and IGF-1R by a nongenomic pathway that was blocked by kaempferol. The results obtained in vitro were confirmed in a breast cancer xenograft mouse model in which the proliferative effect of TCS or E2 was reversed by kaempferol, leading to a reduction of tumor volume [123].

In accordance with the previous results, kaempferol treatment, at the dose of 30 µM, induced apoptosis in MCF-7 (but not in the TNBC MDA-MB-231 cell line) by increasing ROS production that triggered a prolonged state of MEK, ERK1/ERK2, ETS Like-1 protein Elk (ELK) 1 phosphorylation that was not observed in MDA-MB-231 [124]. 

The MEK/ERK pathway that can be activated, for example, by EGF (Epidermal growth factor) also controls RSK2 activity which is responsible for activation of ELK3, a gene positively involved in proliferation and metastatic formation. It was demonstrated that MDA-MB-231 cells treatment with 40 µM of kaempferol led to inhibition of ELK3-mediated gene transcription [125], probably by a direct effect of kaempferol on RSK2 activity (IC_50_ = 15 µM), as demonstrated by an in vitro kinase assay [126]. Another study, in which the cell lines used (T47D, MCF-7, MDA-MB-231 and MDA-MB-468) showed different growth inhibition sensitivity (IC_50_ 123 ± 0.4, 132 ± 0.23, 24.85 ± 0.12 and 25.01 ± 0.11 μg/mL, respectively), highlighted a possible interaction of kaempferol with sirtuins, in particular SIRT1, 2, 3 and 7. This interaction was predicted by in silico stringent analysis and was partly confirmed using the MDA-MB-468 cell line in which kaempferol treatment (IC_50_ = 24.25 μg/mL) decreased SIRT3 protein level [127]. Interestingly, other flavanones proved to be activators of SIRT1 along with AMPK, thus inducing the related axis [22]. Another protein regulated by kaempferol is IQ motif containing GTPase-activating protein 3 (IQGAP3), expressed at high levels in tumor breast tissue. Using ZR-75-30 (ER^+^, PR^−^, HER2^+^) and BT474 (ER^+^, PR^+^, HER2^+^), kaempferol treatment (100 µM maxima concentration) decreased expression of IQGAP3 and phosphorylation of ERK1/ERK2 that correlated with growth inhibition and apoptosis, phenomena that were reversed by IQGAP3 overexpression. The responses observed were the same even if the cell lines used had different genetic characteristics [115]. Using BT474 and MDA-MB-231, another cellular response observed after treatment with 50 µM of flavonol was the induction of apoptosis by a mechanism triggered by a DNA double strand breaks, as demonstrated by increased ATM and γH2AX phosphorylation levels [128].

**Figure 3:**
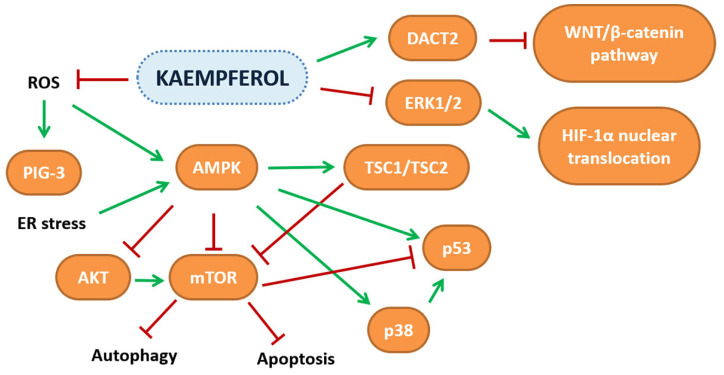
Schematic representation of the pathways affected by kaempferol in several in vitro and in vivo cancer models.

A great problem linked to breast cancer is its tendency to metastasize and great effort is made to find molecules that can reduce this risk. Phromnoi et al., reported that treatment with kaempferol, in addition to growth inhibition of MDA-MB-231 (IC_50_ = 60.0 ± 16.3 µM after 48 h), decreased cell invasiveness, probably caused by direct flavonol-mediated MMP-3 activity inhibition [122]. These results were partly confirmed using the same cell line; however, a higher concentration of kaempferol was necessary to inhibit cell growth (IC_50_ = 204.7 ± 8.9 µM after 24 h) [129]. Kaempferol treatment, at the concentration of 40 µM, led to decreased cellular adhesion, motility and invasiveness by a mechanism in which kaempferol negatively influenced protein kinase C delta (PKCδ) activity, and this was reflected in ERK1/ERK2, p38 and JNK decreased phosphorylation, activator protein 1 (AP-1) cytoplasmic localization, and decreased MMP-9 expression. The anti-metastatic properties of kaempferol obtained in vitro were partially demonstrated also in vivo by administration of a dose of 200 mg/kg of flavonol to an animal model of lung metastasis which reduced the number and volume of nodules and also decreased MMP-9 protein levels [130]. Li et al., in their study, considered cell lines with different molecular characteristics and demonstrated that 20 µM kaempferol treatment of TNBC cell lines led to reduced invasiveness, and migration by Ras homolog family member A (RhoA) and Ras-related C3 botulinum toxin substrate 1 (Rac1) decreased phosphorylation, whereas in SK-Br-3 and in MCF-7 cell lines, the same results were obtained only after co-treatment with kaempferol and herceptin, an human epidermal growth factor receptor 2 (HER2), or AZD9496 and megestrol acetate, a progesterone and an estrogen receptor inhibitors, respectively, although in the last condition, only RhoA phosphorylation decreased. These results highlight that the genetic background of the different breast cell lines must be carefully considered [131]. In MCF-7, whether E2 or TCS stimulated, kaempferol at a concentration of 25 µM reversed both the EMT process and cellular proliferation, as demonstrated by decreased E2 and TCS induced MMP-2, -9, cathepsin B and D protein levels. Although the mechanism was not investigated, it is feasible that the IGF-1R pathway is also involved in this process [132]. The scientific evidence on the anti-cancer activities of kaempferol gathered in this review is reported in Table 2, while the pathways modulated are depicted in Figure 3.

## 4. Conclusions

Given its intrinsic nature, multi-target therapy represents our greatest ally in the battle against cancer. This is where natural products are most effective in providing a response by aiming simultaneously at several targets. In this way, plant-derived compounds can target different stages of the carcinogenic process, and according to an ever-growing body of research, they might be valuable tools for both prevention as well as an adjuvant in anti-cancer therapies. This is corroborated by the fact that these compounds are generally safer and less toxic than the synthetic agents employed in the common therapeutic protocols and are thus better tolerated by patients. For these reasons, more complex studies should be performed to define a complete pharmaco-toxicological profile for natural compounds, such as myricetin and kaempferol, which can truly enhance the current knowledge on anti-cancer therapies and increase hope for patients affected by this nefarious condition.

## Figures and Tables

**Table 1:** Anti-cancer effects of myricetin in different in vitro and in vivo experimental models.

**Hepatocellular carcinoma (HCC)**	**Cell line or animal model**	**Concentration**	**Effect**	**Reference**
	HepG2	33–198 µM	G2/M cell cycle arrest	[42]
	HepG2	20 µM	Increase of pro-apoptotic and decrease of cell cycle progression protein expression; Reduction of preneoplastic nodule formation. Effects related to PAK1 inhibition	[43]
	DEN-Wistar rats animal model	100 mg/Kg		
	HepG2	132–198 µM	Apoptosis induced by decrease of phosphorylated AKT and p70S6K1	[44]
	HepG2, Huh-7	100–200 µM	Apoptosis induced by inhibition of Hippo pathway	[46,47]
	Huh-7-xenograft mice	30 mg/kg/day+ 5 mg/kg/3 days cisplatin	Decrease of tumor growth by apoptosis induction mediated by inhibition of Hippo pathway	
	MHCC97H	100 µM	Inhibition of EMT	[46,47]
**Colorectal cancer (CRC)**	Caco-2	88.4 ± 3.4 µM	Growth inhibition	[58]
	HT-29	47.6 ± 2.3 µM		
	HCT-15	100 µM	Apoptosis induction by a caspase-independent mechanism	[59]
	HCT-15	0–200 µM	Apoptosis induction and metastasis formation inhibition	[60]
	COLO 205	200 µM	No evident effects	[61]
	HCT116	>500 µM	No evident effects	[62]
	HCT116, HT-29, SW480, SW620	0–400 µM	Apoptosis and autophagy induction	[63]
	COLO 205, COLO 320HSR, COLO 320DM, HT-29, COLO 205-X	0–200 µM	MMP-2 protein activity and expression inhibition	[61]
	HT-29	0–64 µM	hFEN1 activity inhibition	[66]
	Caco-2	60 µM flavonol+50 µM oxaliplatin	Reduced MRP2-mediated drug efflux and apoptosis induction	[67]
	APC^Min/+^ mice	100 mg/Kg	Inhibition of adenomatous polyps by cellular growth arrest and apoptosis induction, decrease of inflammation	[71]
	AOM/DSS induced colitis and tumorigenesis mice	100 mg/Kg	Reduced inflammation and tumorigenesis	[72]
**Breast cancer (BC)**	MDA-MB-231	114.75 µM	Growth inhibition by apoptosis induction	[62]
	MDA-MB-468, MCF-7, SK-BR-3	50 µM	Growth inhibition and apoptosis induction by increase of flavonol-mediated ROS production	[73]
	MCF-7	50 µM	Downregulation of *TERT* gene expression	[74]
	MCF-7 and in vitro assay	5–50 µM	Inhibition of telomerase activity by flavonol G-quadruplex binding	[75]
	MCF-7	54 µM	Extrinsic and intrinsic apoptosis induction by BRCA1-GADD45 pathway activation	[76]
	T47D	46 µM	Apoptosis induction by BRCA1-GADD45 pathway activation	[77]
	MCF-7	80 µM	Apoptosis induction by PAK1 decreased expression	[78]
	MDA-Mb-231br	40 µM	Viability inhibition	[81]
		5–10 µM	Migration and invasiveness inhibition by MMP-2 and -9 expression and activity inhibition	
	4T1 mouse lung metastasis model	25 mg/kg	Reduction of tumors number	
	In vitro	7.3 µM	Ornithine decarboxylase activity inhibition	[86]

**Table 2:** Anti-cancer effects of kaempferol in different in vitro and in vivo experimental models.

**Hepatocellular carcinoma (HCC)**	**Cell line or animal model**	**Concentration**	**Effect**	**Reference**
	Huh7	4.75 µM	HIF-1α inhibition	[91]
	HepG2	40 µM	Apoptosis induction by PIG3 upregulation induced by flavonol-mediated ROS increase	[92]
	Hepatocytes derived by HCC rat model	30 µM	Apoptosis induction by flavonol-mediated ROS increase	[93]
	SK-HEP-1	100 µM	Block of cell cycle progression and autophagic cell death induction by AMPK increased activity	[94]
	HepG2, Huh7, BEL7402, SMMC and primary human HCC cells	50 µM	Autophagic cell death induction by AMPK increased activity	[95]
	HepG2	100 µM	Apoptosis induction by ER stress response	[96]
	HepG2, Huh7	100 µM	Apoptosis induction preceded by autophagy	[97]
	Huh-7, SK-Hep-1	100 µM	No cytotoxic effect. Decrease of cellular migration and invasiveness	[98]
	HepG2, N1S1	2.5 µM + 2.5 µM oxaliplatinum	Cell viability decrease	[100]
	Huh7, Huh-1, HepG2, HepG2.2.15, SK-Hep-1, PLC/PRF/5, HLE, HLF, Hep3B	40 µM + 900 nM doxorubicin	Additive effect on reduction of growth, migration and invasiveness, and increase of apoptosis	[101]
**Colorectal cancer (CRC)**	SW480	100 µM	Growth inhibition by apoptosis induction	[102]
	HT29, COLO205, COLO320-HSR, COLO205-X	200 µM	No noticeable growth inhibition	[103]
	SW480	50 µM	Growth and 12(S)-LOX enzymatic activity inhibition	[104]
	DDL-1	40 µM	Inhibition of COX-2 expression	[105]
	SW480	40 µM	Increase of OCTN2 expression	[106]
	SW480, DDL-1	40 µM+ TRAIL ligand	Induction of apoptosis by upregulation of DR5 expression	[107]
	HCT116	0–120 µM	Induction of apoptosis by p53-mediated ATM activation	[108]
	HCT116 *BAX* knock-out, LoVo	100 µM	BAK-dependent apoptosis induction ER stress-mediated	[109]
	HT-29, SW480	0–60 µM	Apoptosis induction by AKT decreased phosphorylation	[111]
	HCT116, HT-29	5 µM	Apoptosis and necrosis increase, cell migration decrease by DNMTs-mediated DACT2 upregulation	[112]
	C57BL/6 mice AOM/DSS CRC induced	150 mg/kg		
	SW480, HCT116, HCT-15	100 µM	Apoptosis induction by p53 and p38/MAPK activity increase mediated by ROS enhanced production	[113]
	LS174-R	75 µM + 5-FU	Decreased cell viability by apoptosis. PI3K/AKT, MAPK, JAK/STAT3 and NF-κB signaling pathway modulation	[114]
	HCT116, HCT-8	100 µM+50 µM 5-FU	Apoptosis induction by PI3K/AKT-mediated decrease of thymidylate synthase protein level	[115]
	HCT116, DDL-1	0–100 µM	Cell viability reduction by miR339-5p-mediated downregulation of PKM2	[116]
	HCT116, LoVo	0–120 µM	Apoptosis induction	[118]
	HCT116	9.427 µM	Cell cycle arrest and apoptosis induction	
	RKO	17.42 µM		
**Breast cancer (BC)**	MCF-7, T47D, ZR-75 (ER^+^)	35 µM	Growth inhibition	[120]
	MDA231 (ER^−^)	70 µM	Growth inhibition by ERα reduced protein level and E2 antagonizing effect	
	VM7Luc4E2	30–40 µM	Apoptosis induction, antagonize E2, bisphenol A and TCS effects by ER stress response triggered by ROS production increase	[122]
	SK-BR-3	100 µM	No apoptosis, autophagy increase	[110]
	MCF-7	50–100 µM	Cell viability decrease by IGF-1R signaling pathway inhibition	[123]
	MCF-7 xenograft mouse model	100 mg/Kg	Tumor volume reduction, apoptosis induction.	
	MCF-7	30 µM	Apoptosis induction triggered by increased ROS production mediated ERK activation	[124]
	MDA-MB-231	0–100 µM	Minor effects on cell viability	
	MDA-MB-231	40 µM	Reduction of cell proliferation and colony formation by ELK3 expression decrease	[125]
	T47D, MCF-7, MDA-MB-231 and MDA-MB-468	12.5–50 µM	Cell viability reduction probably mediated by reduction of SIRT3 protein expression	[127]
	ZR-75-30, BT474	0–100 µM	Apoptosis increase linked to IQGAP3 reduced expression	[128]
	BT474, MDA-MB-231	50 µM	Apoptosis induction mediated by increase of double strand breaks	
	MDA-MB-231	60 µM	Reduction of invasiveness by MMP3 activity inhibition	[129]
	MDA-MB-231	0–40 µM	MMP -9 reduced expression and activity mediated by PKCδ, ERK1/2, p38, AP-1 inhibition	[130]
	MDA-MB-231, MDA-MB-453	20 µM	Migration and invasiveness reduction by RhoA and Rac1expression inhibition	[131]
	SK-BR-3, MCF-7	20 µM+ Herceptin or AZD and MA, respectively	Migration and invasiveness reduction by RhoA downregulation	
	MCF-7	25 µM	Reduced cell proliferation and EMT abilities	[132]

## Data Availability

Not applicable.
